# Infectious Eye Diseases and Prevention Control

**DOI:** 10.3390/microorganisms11051286

**Published:** 2023-05-15

**Authors:** Christopher D. Conrady

**Affiliations:** 1 Department of Ophthalmology and Visual Sciences, Stanley M. Truhlsen Eye Institute, University of Nebraska Medical Center, Omaha, NE 68105, USA; cconrady@unmc.edu; 2 Department of Pathology and Microbiology, University of Nebraska Medical Center, Omaha, NE 68105, USA

Ocular infections are rare but can be unfortunate, vision-threatening conditions that can affect any part of the eye, from the outer tissues including the episcleral, sclera, and cornea to inside the eye such as the anterior chamber, vitreous, optic nerve, and retina. Further, pathogens may affect the space around the eye known as the orbit leading to similar vision-threatening results. With ocular infections, a single tissue may be affected by a pathogen such as in herpetic epithelial keratitis (i.e., cornea), or not uncommonly, multiple tissues may show some reaction as depicted in a panuveitis associated with herpetic retinitis (i.e., anterior chamber and vitreous inflammation with retinal necrosis). To complicate matters, immunological responses within the eye are different from those found in other tissues due to “immune privilege” [1]. This makes the study of the ocular pathogenesis of infectious diseases and the immunological response to these same pathogens important. Unfortunately, in many ocular or periocular infections, our understanding of disease pathogenesis within the eye is lacking inhibiting therapeutic advances despite these relatively poor visual prognoses.

This is epitomized by a blinding, herpetic infection of the retina, acute retinal necrosis. Our current understanding of acute retinal necrosis is limited to the identification of a broad upregulation of chemokines and cytokines within mouse models of the disease and from clinical aqueous or vitreous specimens [2,3]. This disease has been treated in essentially the same manner despite high rates of ocular complications for over thirty years [4,5]. As such, we have developed a mouse model of the disease within our lab to better understand the innate immune response to the virus within the retina to promote bench-to-bedside discoveries [6]. We hope these findings will identify critical pathways and immune responses that can be targeted to promote viral clearance while minimizing neuropathogenesis and may be broadly applicable to the brain in cases of neurologically-devastating, herpetic encephalitis [7].

Within this Special Issue entitled, “Infectious Eye Diseases and Prevention Control”, we hope to advance our knowledge of infectious disease pathogenesis and ocular immunology to identify bacterial targets, neuroimmunological pathways, or potential therapeutics to enhance long-term visual outcomes. We hope to do this by compiling basic science mechanistic studies, reviews, the application of novel preventative strategies, or exceptional case reports within this issue.
